# Introduction of *Surgical Case Reports*: the second official journal of the Japanese Surgical Society

**DOI:** 10.1186/s40792-014-0001-5

**Published:** 2015-01-16

**Authors:** Hideo Baba

**Affiliations:** Department of Gastroenterological Surgery, Graduate School of Medical Sciences, Kumamoto University, 1-1-1 Honjo, Kumamoto-city, 860-8556 Japan

It gives us great pleasure to announce the launch of our new journal, *Surgical Case Reports*: an international peer-reviewed open-access journal, published online by the Japan Surgical Society, in partnership with Springer. This new journal is devoted to original case reports and letters to the editor.

Surgical techniques and perioperative management have advanced rapidly and become more specialized. For example, the archive of robotic surgery is representative of the progress of this exciting breakthrough, which has gained widespread popularity over the last decade. In addition, translational research has come to be a critical and core component of full-spectrum biomedical research and is becoming more important in all medical fields. Large studies on these aspects of surgery, as well as randomized control trials (RCTs) and meta-analyses have provided critical evidence. The guidelines and general rules for the diagnosis and management of various diseases such as malignancy are generally established based on this evidence. However, surgeons are encouraged to report very rare cases, unanticipated surgical complications, or adverse events and explain how they used their resources to resolve them. Surgeons also often report on innovative new ideas to improve procedures in their specialty. Thus, an excellent case report can provide important detailed information about an individual case, which would be lost in an RCT or large study [1]. From case reports, readers can acquire early warning of the adverse effects of new medications or the rare presentations of new and emerging diseases. Whereas much medical and surgical literature includes case reports, these articles are underrepresented in those journals and journals that do publish valuable case reports specifically are very limited. *Surgical Case Reports* fills this void. This exciting new journal is dedicated to the publication of high-quality case reports and aims to expand current knowledge in all surgical fields.

Since 1992, the Japan Surgical Society has been publishing *Surgery Today* as its official journal. *Surgery Today* is a monthly journal, with an archive of 44 volumes to date. Because *Surgery Today* is published in English and documents the latest progress in all surgical fields, articles in this journal have been widely indexed. The number of submissions to *Surgery Today* has increased steadily to more than 1,200 per year; however, as the number of pages is limited, so is the number of papers that can be published after careful peer review. Until now, *Surgery Today* has included original articles, review articles, short communications, ‘how-to-do-it’ papers, letters to the editor, and case reports. It has been decided that the focus of *Surgery Today* will be original articles, so that more papers documenting recent surgical progress can be communicated to the world in a timely manner. Hence, we decided to establish a new journal: *Surgical Case Reports* [2]. The purpose of this journal is to contribute to the progress of surgery by providing clinicians and researchers with an educational forum. This journal will disseminate each contributor's personal experience and novel treatments to a wide readership and share interesting rare cases encountered by colleagues all over the world.

*Surgical Case Reports* welcomes well-described reports of cases, which include the following:

- ✓ New associations or variations in disease processes.
- ✓ Presentations, diagnoses, and/or management of new and emerging diseases.
- ✓ Unreported or unusual side effects or adverse interactions involving medications.
- ✓ Unexpected or unusual presentations of a disease.
- ✓ An unexpected association between diseases or symptoms.
- ✓ An unexpected event in the course of observing or treating a patient.
- ✓ Findings that shed new light on the possible pathogenesis of a disease or an adverse effect.
- ✓ Movies of the author's surgical teaching or technical development.

*Surgical Case Reports* also invites letters to the editor, which generally take one of the following forms:

- ✓ A substantial re-analysis of a previously published article in *Surgical Case Reports* or another journal.
- ✓ An article that may not cover ‘standard research,’ but is of general interest to the broad readership of *Surgical Case Reports*.
- ✓ A brief report of research findings adequate for the journal's scope and of particular interest to the community.

All manuscripts submitted to *Surgical Case Reports* will be subject to immediate screening by the in-house editorial team and selected manuscripts will be sent for peer review. Accepted articles will be published online and indexed in PubMed.

Open-access is a modern system of publication, which provides a valuable opportunity for both readers and authors to expose and share the publications. Based on this new form of publication, we anticipate that *Surgical Case Reports* will become a useful interdisciplinary journal.

We hope that we have aroused your interest and that you will support this exciting new journal by submitting your next case report to *Surgical Case Reports*.
